# Hemostatic agents for prehospital hemorrhage control: a narrative review

**DOI:** 10.1186/s40779-020-00241-z

**Published:** 2020-03-25

**Authors:** Henry T. Peng

**Affiliations:** Defence Research and Development Canada, Toronto Research Centre, 1133 Sheppard Avenue West, Toronto, ON M3K 2C9 Canada

**Keywords:** Hemostatic agent, Hemorrhage control, Trauma

## Abstract

Hemorrhage is the leading cause of preventable death in combat trauma and the secondary cause of death in civilian trauma. A significant number of deaths due to hemorrhage occur before and in the first hour after hospital arrival. A literature search was performed through PubMed, Scopus, and Institute of Scientific Information databases for English language articles using terms relating to hemostatic agents, prehospital, battlefield or combat dressings, and prehospital hemostatic resuscitation, followed by cross-reference searching. Abstracts were screened to determine relevance and whether appropriate further review of the original articles was warranted. Based on these findings, this paper provides a review of a variety of hemostatic agents ranging from clinically approved products for human use to newly developed concepts with great potential for use in prehospital settings. These hemostatic agents can be administered either systemically or locally to stop bleeding through different mechanisms of action. Comparisons of current hemostatic products and further directions for prehospital hemorrhage control are also discussed.

## Background

Hemorrhage, in particular noncompressible hemorrhage from the splanchnic and junctional regions, remains the leading cause of preventable death on the battlefield [1, 2]. In the United States (US) conflicts in Afghanistan and Iraq from October 2001 to June 2011, 90% of combat casualties died prior to arrival at surgical facilities, and approximately 90% of potentially survivable deaths were due to hemorrhage, with 67, 19 and 14% from truncal locations, junctional locations and extremities, respectively [2, 3]. In civilian practice, traumatic injury is the second leading cause of death, accounting for 10% of deaths worldwide [4], with up to 40% mortality due to hemorrhage [5]. In 2013 in the US, trauma was associated with an economic cost of approximately $670 billion in medical care expenses and lost productivity [6].

Coagulopathic bleeding is frequently present early after major trauma (one in four patients at admission), with a three- to five-fold increase in mortality [7, 8]. In addition, a significant number of deaths due to hemorrhage occur in the first several hours after injury in both civilian and military trauma [9]. Most in-hospital deaths occur in the first hour after hospital arrival [10]. Therefore, hemostatic agents for prehospital and early in-hospital administration are particularly important. As a result, there has been a recent interest in hemostatic resuscitation in prehospital settings, the so-called ‘remote hemostatic resuscitation’ [11, 12].

Significant advances in hemorrhage control and hemostatic resuscitation have been made with the emergence of a new paradigm referred to as ‘damage control resuscitation’ [13]. Transfusion of existing blood products (i.e., red blood cells, fresh frozen plasma, platelets) for trauma has been conducted and has been shown to be logistically achievable without serious incident in prehospital settings, although no definitive conclusions as to any improved outcomes can be made given the low level of evidence [14–17]. In addition, new hemostatic agents, surgical adjuncts and blood products have been developed for prehospital control of life-threatening noncompressible hemorrhage from the splanchnic and junctional regions [14, 18–20]. Approximately 47% of respondents in Emergency Medical Services indicated they carry hemostatic products such as Combat Gauze [21]. All these measures may have led to reduced mortality from battlefield and civilian trauma.

Ideal hemostatic agents for battlefield and prehospital hemorrhage control should have the following characteristics [22–24]:

1. Quick and effective control of bleeding in a wide range of conditions and from a variety of wounds within 2 min, even when applied to an actively bleeding site through a pool of blood;
2. Sustainable hemostasis duration for several hours if used on the battlefield, reflecting delayed evacuation;
3. Easy removal without leaving residues or no need for removal as a result of biodegradation; ready to use with little training and preparation;
4. Easy administration even by a layperson under austere conditions;
5. Ease of manufacture and sterilization and low cost;
6. Simple storage and high portability; prolonged stability (>two-year shelf-life), even under extreme conditions (− 10–55 °C);
7. Good biocompatibility with no adverse effects on healing and no thromboembolic complications.

The criteria for material selection should be based on probability of success in vivo, stability, ease of use, and ease of manufacturing.

No current hemostatic agents meet all the requirements. There are continuous efforts to develop new hemostatic agents with various delivery mechanisms for treating severe hemorrhage in a military setting. These include self-propelling particles [25], hydrophobically modified chitosan gauze [26], injectable and in situ forming gels [27, 28], and self-expanding foams [29]. Given the safety concerns with some currently used hemostatic materials [30, 31], more efficient and safer hemostatic agents are still needed.

This review will focus on those that have been approved for human use on the battlefield or have potential for hemorrhage control in prehospital settings. The topic is discussed according to the routes of application for hemorrhage control: systemic versus local. Current hemostatic agents possess different biochemical properties and mechanisms of action. The primary types of hemostatic agents reviewed in this article are dried plasma, fibrinogen concentrate (FC), tranexamic acid, dried and synthetic platelets, and topical hemostatic products currently deployed in combat and prehospital settings (Combat Gauze, HemCon, Celox, XStat). Both preclinical and clinical studies have been conducted to compare the hemostatic efficacies of these agents. In addition, future research into hemorrhage control with hemostatic agents will be discussed.

## Methods

A broad PubMed search without restriction to publication date for English language articles relating to hemostatic, battlefield or combat dressings and/or prehospital hemostatic resuscitation was performed and followed by cross-reference searching. The search terms were as follows: prehospital or pre-hospital or battlefield or combat or military, trauma* or wound or bleeding, hemorrhag* or haemorrhag*, or hemostatic agent or dressing. The specific product names and active agents (e.g., Combat Gauze: a kaolin-impregnated nonwoven gauze made by Z-Medica Corporation; HemCon, ChitoGauze, and ChitoFlex: chitosan-based dressings made by HemCon Medical Technologies; Celox: granules and gauze made from more than one type of chitosan and manufactured by Medtrade Products Ltd.; XStat produced by Revmedx, and chitosan, dried plasma, fibrinogen concentrate, fibrin sealant, tranexamic acid, platelet) were also used as keywords in the search.

Abstracts were screened to determine relevance and when appropriate further review of the original articles was warranted. Additional publications were selected from the cross-references listed in the original papers and from the cited articles, and additional searches were performed through Medline, Scopus and Institute of Scientific Information databases for those topics with limited findings from PubMed.

The search was primarily focused on human studies of the hemostatic agents already operationally deployed for control of severe/active bleeding or deemed to be applicable in both military and civilian prehospital settings. Some animal studies that employed lethal injury models for comparison of combat and prehospital hemostatic agents were included given the limited number of comparative studies in humans. All human studies were eligible for inclusion, regardless of study design (i.e., trials, case reports and reviews). The focus of this review was on hemostatic agents and thus hemostatic devices (e.g., tourniquets) were excluded.

It should be noted that the literature search, screening and assessment were not conducted in accordance with Preferred Reporting Items for Systematic Reviews and Meta-Analysis (PRISMA) protocol [32]. As the search was performed by a single author, there may be bias for the assessment of relevance of articles for inclusion. The review was limited to English language papers with dominance of local hemostatic agents and several dressings.

## Systemic hemostatic agents

Systemic hemostatic agents can be divided into blood products or synthetic materials. The former includes fresh frozen plasma (FFP), dried plasma, platelets and coagulation factor concentrates, while the latter includes synthetic platelets, polymers and composite materials (Table 1).

**Table 1:** Potential hemostatic agents for prehospital and combat hemorrhage control

Typical hemostats	Bleeding sites	Comments
Intravenous infusion		
Coagulation factor concentrates: fibrinogen, recombinant factor VII, prothrombin complex [33–36]	Extremity/junctional/truncal hemorrhage	Used clinically and in remote operational environments, showing logistic benefits, requiring more randomized controlled trials for clinical benefits
Dried plasma [37, 38]	Extremity/junctional/truncal hemorrhage	Used prehospitally and on the battlefield, showing logistic benefits and a positive effect on coagulation profile, with no effects on other outcomes
Tranexamic acid [39–42]	Extremity/junctional/truncal hemorrhage	Used prehospitally and on the battlefield, suggesting a survival advantage to severely bleeding patients
Dried platelets [43–45]	Extremity/junctional/truncal hemorrhage	Under development
Platelet substitutes/synthetic platelets [46–48]	Extremity/junctional/truncal hemorrhage	Under development
Synthetic polymers: polySTAT [49–51]	Extremity/junctional/truncal hemorrhage	Under development, improved survival compared to an albumin control in a rat femoral artery injury
Local application		
Hemostatic dressings: Combat Gauze, Celox Gauze, ChitoGauze, HemCon dressing [52–54]	Extremity/junctional hemorrhage	Used on the battlefield and in prehospital settings
Injectable and self-expanding sponges (XStat), intracavitary forms (ResQFoam, ClotFoam), in situ forming gels, self-propelling particles [19, 25, 27, 55, 56]	Truncal hemorrhage	Under development

### FFP

Systemically, early and aggressive hemostatic therapy has been shown to improve survival in combat casualties [57]. Blood products and coagulation factor concentrates are systemically used for hemostatic resuscitation in trauma [58]. FFP is the hemostatic agent used to restore lacking coagulation factors but is not ideal for use in far-forward combat environments and prehospital settings for logistics reasons e.g., requirements for freezers and thawing equipment. However, two recent randomized controlled trials have demonstrated the technical and logistical feasibility of prehospital use of FFP in severe trauma, although conflicting results on its efficacy have been reported [59, 60]. The study conducted in 501 trauma patients at risk for hemorrhagic shock during air medical transport showed a lower 30-day mortality rate in the FFP compared to the standard care group (23% vs. 33%; *P* = 0.03) [59]. In contrast, the study comparing FFP versus normal saline in 144 trauma patients with hemorrhagic shock during emergency ground transportation found no difference in mortality at 28 days between the two groups (15% vs. 10%; *P* = 0.37) [60]. The discrepancy might be due to different characteristics between the two trials (e.g., injury severity) [17].

Alternatively, lyophilized plasma [61] and fibrinogen concentrate [62] are promising systematic hemostatic agents for hemorrhage control on the battlefield given their long storage stability, easy carry-on and fast reconstitution. Although these agents have technical and logistic superiority over allogenic blood products (e.g., FFP, cryoprecipitate and whole blood), further studies are needed to determine the optimal hemostatic resuscitation strategy [33, 63].

### Prothrombin complex concentrate (PCC)

It is not clear whether PCC can be effective as an adjunct in patients who require massive transfusion [34]. PCC is a plasma-derived factor concentrate containing coagulation factors II, VII, IX, X and anticoagulant proteins C and S. In addition to four-factor PCC, three-factor PCC, which does not include factor VII, is available [64]. The major advantages of PCC are the high concentration of factors in a small volume and the potential benefit of rapid access and timely treatment in either prehospital care or emergency admission [65]. Although retrospective and prospective studies of PCC (±FC) have demonstrated a decreased time to correction of trauma-induced coagulopathy and decreased red cell transfusion with no obvious effects on mortality or thromboembolic outcomes, more prospective, randomized, controlled trials need to be undertaken for its use as a first-line therapy for patients with trauma-related bleeding [66].

### Dried plasma

Three different products of dried plasma are commercially available [37, 67]: French lyophilized plasma (FLYP) manufactured by the French Blood Bank from approximately 10 carefully screened and monitored donors; German lyophilized plasma (LyoPlas) manufactured by the German Red Cross from a single donor quarantined for at least 4 months and negatively tested for human immunodeficiency virus and hepatitis B and C virus; and Bioplasma freeze-dried plasma (FDP) produced by the National Bioproducts Institute of South Africa from hundreds of donors (up to 1500). In addition to the source of plasma, FLYP incorporates pathogen reduction using an amotosalen and ultraviolet light process, while the latter two use solvent-detergent technologies for pathogen reduction.

FLYP and LyoPlas have been recommended for hemorrhage control in combat and prehospital settings. For example, Israel Defense Forces have successfully used LyoPlas at the point of injury in the battlefield [61, 68], and a retrospective study has shown the feasibility and safety of LyoPlas for civilian prehospital hemorrhagic shock resuscitation [69]. Another recent retrospective study demonstrated that prehospital transfusion of LyoPlas alone or with packed red blood cells (RBCs) in a 1:1 ratio was feasible in patients with suspected traumatic hemorrhage and that prehospital use of the freeze-dried plasma reduced the transfusion of packed RBCs and the time to transfusion [70]. A randomized open-label trial showed that FLYP reduced the transfusion time and achieved a higher fibrinogen concentration and a greater improvement in prothrombin time, factor V and factor II compared to FFP in trauma patients [71]. A recent retrospective study showed that FLYP enabled faster plasma transfusion compared to FFP and a more rapid increase in the plasma-to-RBC transfusion ratio [72]. A case report showed successful intraosseous administration of LyoPlas for prehospital resuscitation of a 13-year-old girl suffering from severe hemorrhagic shock as a result of gunshots and grenade blast [73]. A retrospective matched cohort study based on two groups of casualties (those treated with dried plasma vs. those without the treatment) showed that the use of dried plasma in the prehospital setting has logistic benefits and a positive effect on coagulation profile, with no other significant effects [38].

Sufficient data support further clinical studies on FLYP and LyoPlas for prehospital use [74, 75]. In addition, there are a number of dried plasma products under development [37, 76]. The challenges to the development and use of dried plasma in trauma and critical care involve regulatory issues, logistics, product issues, implementation and commercial viability [67].

### Fibrinogen concentrate

Fibrinogen is perhaps the most important protein in hemostasis, as the final stage of the coagulation cascade, and is converted to fibrin by thrombin and crosslinked by factor XIII. It also induces platelet activation and aggregation via binding to glycoprotein GPIIb/IIIa receptors on the surfaces of platelets, acting as the bridge for stable clot formation [77]. During major bleeding, fibrinogen is the first clotting factor to reach critically low levels below the normal physiological level of approximately 2 to 4 g/L, which is associated with increased bleeding, coagulopathy, and, in turn, worsened clinical outcomes [78–81].

FC is derived from human plasma and is commercially available as different products: Haemocomplettan or RiaSTAP in the USA and Canada (CSL Behring, Marburg, Germany), Clottafact (LFB, Les Ulis, France), Fibrinogen HT (Benesis, Osaka, Japan), and FibroRAAS (Shanghai RAAS, Shanghai, China) [82]. Fibryga (Octapharma, Lachen, Switzerland) is a new highly purified FC [83]. In vitro and clinical studies showed a higher factor XIII level in Fibryga (10.1 vs. 7.2 U/ml) [84], a slower clearance (0.665 vs. 0.804 ml/kg/hr) and a larger volume of distribution (70.158 vs. 76.631 ml/kg) for Fibryga than RiaSTAP, respectively [85]. In contrast, another clinical study reported even smaller clearance (0.53 ml/kg/hr) and volume of distribution for Clottafact (50.7 ml/kg) [86].

Several retrospective studies and case series have reported improved outcomes using FC in trauma [62, 87]. There have been some debates about its clinical benefits [88, 89] and its use as a universal hemostatic agent [90]. Very recently, there have been a few randomized controlled trials of FC as a pre-emptive first-line treatment for trauma hemorrhage in prehospital [91] and early hospital settings [35, 92]. As these studies were focused on feasibility and safety with a small number of patients, they did not show any clinical benefits. The latest randomized trial of first-line therapy using FFP or coagulation factor concentrates (CFC) showed that CFC (primarily FC) is superior to FFP [93]. However, as fibrinogen deteriorates before other coagulation parameters, such as prothrombin time, partial thromboplastin time, and platelet count, and before the need for massive transfusion in the early phase of severe trauma [94], fibrinogen replacement may be required in prehospital settings or at hospital admission to provide clinical benefits. Accordingly, the Canadian Armed Forces adopted FC (RiaSTAP, CSL Behring) for damage control resuscitation in the far-forward combat setting [36]. Moreover, a prospective, randomized, placebo-controlled, double-blinded trial demonstrated that prehospital administration of FC (Clottafact, LFB France) is feasible, protects against early fibrinogen depletion and promotes rapid blood clot initiation and clot stability in trauma patients [95].

### Tranexamic acid

Tranexamic acid (TXA) is a synthetic antifibrinolytic drug derived from the amino acid lysine. It competitively inhibits the activation of plasminogen to plasmin and at higher concentrations and noncompetitively inhibits plasmin from breaking down fibrin clots [96]. TXA also blocks the binding of α-2 antiplasmin to plasmin to prevent plasmin activation. Thus, rather than promoting new clot formation, TXA hinders fibrinolysis to reduce bleeding. In addition, recognizing that many cells of the immunoinflammatory response to stress contain plasminogen receptors on their surfaces, it is conceivable that TXA may have beneficial effects in injured patients independent of the regulation of fibrinolysis [97]. An in vitro study showed that TXA reduced fibrinogenolysis in addition to the correction of fibrinolytic effects in the presence of tissue plasminogen activator [98].

It has been reported that TXA reduces mortality in trauma patients and seems to be most beneficial if given within 3 hours after injury [99]. In the past few years, TXA has drawn much attention for prehospital use in military [39, 100–102] and civilian practice [40]. Overall available data in the literature, including CRASH-2 (Clinical Randomisation of an Antifibrinolytic in Significant Hemorrhage 2) [103] and the WOMAN Trial (World Maternal Antifibrinolytic Trial) [104], corroborate the efficacy and safety of TXA. Literature data also suggest early use of TXA in various clinical settings to reduce blood loss irrespective of the type of surgery and bleeding volume for providing a survival advantage to many patients [105]. A meta-analysis of the randomized controlled trials for the efficacy of prehospital administration of TXA in trauma patients showed a trend toward lower 30-day mortality and a reduced risk of thromboembolic events [41].

TXA is usually given intravenously. Development of a means for intramuscular injection, such as an autoinjector, would allow for immediate treatment on the battlefield where establishing IV access and controlled infusion of drugs is difficult and may be practically impossible. Other routes of administration, e.g., oral and local delivery to bleeding sites, have been investigated to achieve a prolonged anti-fibrinolytic effect [106]. There is also evidence that topical TXA administration reduces surgical bleeding [107] and is as effective as intravenous infusion [108]. These findings imply that TXA reduces bleeding even in patients without hyperfibrinolysis.

A minimal concentration of 5 mg/L TXA in the blood was determined to have an antifibrinolytic effect [109]. TXA has been incorporated in the resuscitation protocols of severely bleeding patients in many trauma centers [110]. Prehospital TXA use may be selective for acutely injured patients with severe hemorrhagic shock or traumatic brain injury [111] and optimally guided by viscoelastic hemostatic assays [97].

### Intravenous/infusible hemostats under development

In addition to the aforementioned systemic hemostats currently in clinical use, there are a few biological and synthetic agents at various stages of development for potential use on the battlefield and in the prehospital environment, as described below.

#### Dried platelets

Given the essential role of platelets in hemostasis, transfusion of platelet concentrates is an important component of hemostatic resuscitation for treatment of traumatic hemorrhage [112]. Platelet concentrates can be produced from either whole blood or by apheresis and can be stored at room temperature for 5 to 7 days. Thus, the ability to store platelets until times of scarcity is appealing under austere prehospital environments.

Human lyophilized platelets that can be prepared for intravenous infusion in 5–10 min by the addition of sterile water may provide the combat medic and civilian first responder an easy-to-use and effective treatment to reduce blood loss from noncompressible hemorrhage at all levels of care and may represent a promising hemostatic agent. However, with the exception of two limited human studies conducted over 50 years ago [113, 114] and one recent safety study in normal subjects [115], there have only been animal studies performed assessing the safety and hemostatic efficacy of lyophilized platelets [43, 44, 116]. For example, lyophilized platelets called Stasix (Entegrion, Research Triangle Park, NC, USA) were produced by a proprietary process involving the covalent cross-linking of surface membrane proteins and lipids that allows stabilization for lyophilization and results in a state of partial activation [117]. An initial in vivo evaluation of the lyophilized platelets in noncompressible porcine liver injury model showed improved survival, reduced blood loss, and evidence of thrombi on necropsy compared to normal saline [45]. However, another study showed that the platelet-derived hemostatic agent could neither reduce ear bleeding time nor improve clot strength in a rabbit thrombocytopenic model [117]. The limited efficacy, immunogenicity and risk of pathogen infection prompt the development of new intravenous hemostats, including synthetic platelets and polymers [46, 49], and platelets loaded with liposome-encapsulated thrombin [118].

#### Synthetic platelets

Due to limited efficacy and portability, the need for antigen matching, high risk of bacterial contamination, and a short shelf-life of donor-derived platelets, a variety of platelet-like intravenous synthetic agents have been developed [47, 48].

Micro/nanosized synthetic liposomes and particles were surface-modified by platelet-binding motifs or platelet surface adhesion peptides to mimic certain components of platelet adhesion, aggregation, and activation, and the formation of a stable hemostatic plug [119]. These synthetic platelets have demonstrated improved hemostasis in animal models of bleeding. For example, liposomes decorated with various binding peptides could reduce bleeding time by 50% in a mouse tail transection model [120]. Collagen and von Willebrand factor-binding peptides were incorporated to mimic platelet adhesion, while a fibrinogen-mimetic peptide was incorporated to promote platelet aggregation. Such synthetic platelets called SynthoPlate were found to reduce blood loss compared to controls (unmodified liposomes and normal saline) in a mouse liver injury model with uncontrolled intraperitoneal hemorrhage [121].

Lavik’s group developed nanoparticles made with a biodegradable poly (lactic-co-glycolic acid) block copolymer core and polyethylene glycol arms terminated with RGD peptides. RGD is a platelet-binding peptide and can interact with activated platelets via the glycoprotein IIb/IIIa receptor [122, 123]. In a mouse model of blast trauma with multiple organ hemorrhage, intravenous administration of the hemostatic nanoparticles led to a significant improvement in survival over the short term (1 h post-blast), with no complications out to 3 weeks [123]. The synthetic hemostats using a poly (lactic acid) core instead of poly (lactic-co-glycolic acid) were stable at 50 °C for 7 days and were still effective at stopping bleeding and improving survival over the 1-h time period in a rat liver injury model [122]. This increased stability would allow for use at extreme temperatures both prehospital and in the combat field. The latest results in a large swine model of liver injury demonstrated that hemostatic nanoparticles were capable of rapidly stopping bleeding within 10 min of administration, even though some signs of vasodilation were still seen [124]. It would be meaningful to compare these new hemostatic agents with existing clinically used ones (e.g., FC).

Welsch et al. reported different platelet-like particles carrying polyethylene glycol side chains and fibrin-targeting single-domain variable fragment antibodies [125]. In vitro studies showed that the platelet-like particles were comparable to natural platelets with regards to enhancing the fibrin network formation through strong adhesion to the emerging fibrin clot and physical, noncovalent crosslinking of nascent fibrin fibers. The particles also enhanced the stability of the fibrin clot against plasmin-induced fibrinolysis. Further studies are warranted to investigate the potential of designed platelet-like particles for use as hemostatic agents in emergency medicine and prehospital settings.

#### Synthetic polymers

The intravenous synthetic polymers and nanoparticles using specific targets and elements for the coagulation cascade are intriguing given their low cost and long stability.

PolySTAT is made of a linear poly (hydroxyethyl methacrylate) (Mw = 45 kD) back grafted with ~ 16 fibrin-binding peptides (Ac − Y (DGl) C (HPr)YGLCYIQGK-Am) [126]. In vitro and in vivo studies have shown its beneficial effects on clot formation by cross-linking fibrin and potential use in transfusion for the treatment of coagulopathy and hemorrhage control in a rat femoral artery injury model [50, 126]. A recent study showed the binding and augmentation of clot formation through multivalent display of the fibrin-binding peptides along the polymer backbone and the optimal valency of four peptides per polymer for maximum hemostatic function [51]. In addition, when loaded onto a nonwoven chitosan gauze, the PolySTAT/chitosan composite gauze reduced blood loss and improved the survival rate compared to a commercial hemostatic gauze (Celox Rapid) in a rat model of femoral artery injury [127].

Another type of synthetic polymer was prepared via conjugation of a glutamine-containing peptide derived from α2-antiplasmin to 8-armed polyethylene glycol [128]. In vitro tests showed that the polymer-peptide conjugate could target factor XIIIa during clotting to form stable and adhesive clots. Moreover, together with spermidine as an amine donor, the conjugate increased the adhesive strength of clots in FXIII-supplemented normal plasma to excised blood vessels by over 2-fold and maintained the strong adhesion under fibrinolytic conditions. Further tests in whole blood from patients with trauma-induced coagulopathy are required for development of this hemostatic agent.

Several synthetic peptides have been developed as injectable hemostatic agents based on different mechanisms. Amphiphilic peptides containing a self-assembling sequence and tissue factor (TF)-binding sequence were synthesized by a solid-phase method [129]. The peptide with the TF- binding sequence of RTLAFVRFK reduced blood loss by 53% versus sham in a rat model of liver injury.

Polyelectrolyte multilayer microcapsules were developed for targeted delivery of hemostatic agents (e.g., FVIII) [130]. The microcapsules can hybridize with platelets and remain intact during circulation upon intravenous administration. Only at the site of vascular injury, the hybridized platelets activate and exert contractile force to rupture the microcapsules and release hemostatic agents to bleeding sites. In vivo studies are needed to demonstrate the targeting mechanism and hemostatic efficacy.

Systemic administration of hemostatic agents requires intravenous access and preparation time (rewarming refrigerated liquid products or reconstituting solid hemostats), which imposes challenges in austere battlefield and prehospital settings. Alternatively, local administration of hemostatic agents may decrease the time to treatment and reduce systemic adverse effects (e.g., thromboembolism), but may be complicated in severe bleeding as a result of the outward flow of blood, which prevents therapeutics from reaching the damaged vasculature [131].

## Local hemostatic agents

The past decade has seen tremendous development of local/topical hemostatic agents for hemorrhage control in prehospital settings [20, 132, 133] as well as in trauma and emergency surgery [134, 135]. The latest hemostatic products and methods include [136] Combat Gauze (Z-Medica Corporation, Wallingford, CT, USA; http://www.z-medica.com/), Celox gauze (Medtrade Products Ltd., Crewe, UK; http://www.celoxmedical.com/usa/products/celox-gauze/) and ChitoGauze (HemCon Medical Technologies, Portland, OR, USA; http://www.hemcon.com/). These hemostatic dressings have been recommended in the US military Tactical Combat Casualty Care guidelines [52, 53] and have been covered in a recent systematic review [54]. HEMO-fiber gauze (CoreLeader Biotech Co., Ltd., New Taipei City, Taiwan, China; www.coreleaderbio.com) has emerged as an alternative. A retrospective review of prehospital applications of chitosan- (HemCon gauze, Celox gauze) and kaolin-based (Combat Gauze) hemostatic agents in Iraq and Afghanistan showed that only 0.9% patients had documented hemostatic use, with more frequent use in casualties with gunshot wounds, higher injury severity scores, traumatic amputations, concomitant tourniquet application, and greater blood product administration [137].

Although most local hemostatic agents are in the form of gauze and membrane sheets, hemostatic agents in particulate, sponge, foam, and gel forms have been developed for treating deep penetrating wounds and intracavitary blunt injuries. New potential products are emerging, such as self-propelling particles [25, 138] and injectable gels [27, 28], self-expanding forms [29], and expanding minisponges [139].

### Combat gauze

This kaolin-impregnated gauze acts as both a factor concentrator by absorbing blood liquid and a procoagulant by initiating the coagulation cascade. Initially developed and approved for military use, prehospital use of the topical dressings by Israel Defense Forces Medical Corps was successful for junctional and extremity hemorrhage control at the point of injury [140]. However, the evidence was based on lower-level human (case series and reports) and animal (swine models with various vascular injuries) studies. A multi-institutional retrospective analysis of clinical outcomes following prehospital use of Combat Gauze found that it was 89% effective for hemorrhage control and associated with minimal morbidity in rural civilian trauma across a wide range of wounds on the head or face, upper and lower extremities, and junctional regions [141]. A prospective report of 30 prehospital uses of Combat Gauze in a civilian setting showed 73% cessation of bleeding and 20% reduced bleeding [142]. In a swine model of high-grade hepatic injuries and hypothermic coagulopathy, animals packed with Combat Gauze had less blood loss and resuscitation requirements when compared with plain laparotomy pads [143].

Limited data on Combat Gauze use for trunk hemorrhage in human patients suggest less efficacy than for extremity (upper and lower limbs) and junctional (head/neck/face) hemorrhage (78% vs. 90 and 100% cessation of bleeding) [144]. A retrospective review compared clinical outcomes of patients receiving intra-abdominal packing with laparotomy pads alone and both pads and Combat Gauze during damage control laparotomy. The review found no additional benefit or morbidity identified with its use compared to standard packing [145]. High-level human studies, e.g., large-scale randomized controlled trials are required to determine the effectiveness of Combat Gauze, in the management of trauma casualties in the prehospital setting [146]. Combat Gauze is the primary hemostatic dressing currently fielded for all US Operational Forces and North Atlantic Treaty Organization (NATO) militaries.

### Chitosan-based hemostatic agents

Chitosan is a widely used biopolymer with unique bioactivities, including hemostatic activities [147]. A number of chitosan-based hemostatic products have been developed for the US military and have gone through a number of generations [52, 148]. These products are manufactured in the forms of wafer, roll and granule. The current generations include Celox RAPIT and Celox Gauze made by MedTrade products Ltd. (Crew, UK) and ChitoGauze made by HemCon Medical Technologies (Portland, USA).

Since chitosan works primarily via direct electrostatic interaction between its positively charged amino groups and the negatively charged cell membranes of erythrocytes, independent of the host coagulation pathway, chitosan-based hemostatic products may also be able to stop coagulopathic bleeding [148]. Extensive evaluations in large animal models of severe bleeding have been conducted to demonstrate their hemostatic efficacy and safety, mostly in comparison with Combat Gauze [149]. Clinical case reports strongly suggest the effectiveness of Celox Gauze when applied in the prehospital setting [150]. A randomized controlled trial showed that Celox Gauze achieved hemostasis (time to hemostasis and blood loss) more effectively than standard pressure dressing in patients with penetrating limb trauma [151]. A prospective study of 66 cases of prehospital use of ChitoGauze showed approximately 70% cessation of hemorrhage and 20% reduced hemorrhage with no observed side or adverse effects in massive traumatic bleeding in civilian emergency medical services [152]. The efficacy of ChitoGauze was not influenced by coagulopathy.

In addition to hemostasis, chitosan exhibits anti-bacterial activity and can be combined with other materials for hemostatic dressings [148].

### XStat

The latest XStat (Revmedx, Wilsonville, OR, USA) is composed of a multitude of rapidly expanding minisponges loaded in a syringe. It can be injected into deep wounds, then quickly expands and creates an internal compression to achieve hemostasis. The sponges are not biodegradable and need to be removed, and each has a tiny radiopaque marker so that any remaining in the body can be spotted on X-ray.

A comparison between XStat and Combat Gauze was conducted in a coagulopathic swine model of lethal junctional hemorrhage, suggesting better hemostasis (faster time to reach hemostasis and less blood loss) in the XStat-treated group [153]. XStat has now been approved by the Food and Drug Association and recommended by the Committee on Tactical Combat Casualty Care as an adjunct for the management of junctional hemorrhage (i.e., bleeding from the neck, the axilla, and the groin) [55]. Clinical studies demonstrated that it was safe and could be rapidly deployed to provide a high degree control of hemorrhage from cavitary wounds not amenable to tourniquet placement in penetrating trauma [154]. One drawback is the removal of the sponge material, which should always be followed by radiographic clearance.

### Fibrin sealant patch

Different solid fibrin sealant products have been developed under commercial names: TachoSil [155], TachoComb [156, 157], fibrin pad [158, 159], dry fibrin sealant dressing [160–163] and Fibriseal [164]. Table 2 summarizes a variety of fibrin sealant patches/dressings. These products differ in their exact compositions (e.g., fibrinogen and thrombin concentrations), physical characteristics (e.g., backing materials) and types of thrombin (i.e., bovine plasma-derived thrombin, human plasma-derived thrombin, and human recombinant thrombin [166]). This may explain the different effectiveness in in vivo and clinical studies. In addition, thrombin-coated polyglycolic acid sheet (75 U/cm^2^) coupled with liquid fibrinogen (20 mg/ml) was sufficient to achieve hemostasis in a canine model of pulmonary arterial hemorrhage [167].

**Table 2:** Commercial fibrin sealant patch/dressings

Product name	Composition	Applications
TachoSil (Takeda Pharma A/S) [155]	An absorbable fibrin sealant patch consisting of a thin equine collagen sponge coated with human fibrinogen (5.5 mg/cm^2^) and human thrombin (2.0 U/cm^2^)	Secondary treatment of local bleeding in patients undergoing hepatic resection
Fibrin sealant (TachoComb H) [156]	Equine collagen layered with human fibrinogen, 5.5 mg/cm^2^, and human thrombin 2.0 U/cm^2^	Applied to cortical brain lesions in rabbits under normal coagulation and following exposure to plasminogen activator to induce hyperfibrinolysis
Fibrin sealant dressing (TachoComb) [157]	Equine collagen layered with human fibrinogen, 4.3–6.7 mg/cm^2^, and bovine thrombin 1.5–2.5 U/cm^2^, Hafslund Nycomed Pharma	Applied to the cut surface of a rat kidney with light digital pressure for 2 min. After release of digital pressure, the dressing remained on the kidney for 30 min
Fibrin Pad (Omrix Biopharmaceuticals Ltd.)	A sterile, bioabsorbable product consisting of 2 constituent parts: a flexible matrix consisting of polyglactin (PG910) filaments needle-punched into a backing fabric of oxidized regenerated cellulose and a coating of human fibrinogen (7.8 mg/cm^2^) and human thrombin (31.5 U/cm^2^)	Management of soft tissue hemorrhage occurring during major surgical operations in the abdomen, retroperitoneum, pelvis, and noncardiac thoracic surgery [158]
		Used as an adjunct to achieve hemostasis during hepatic surgery [159]
Dry fibrin sealant dressing (DFSD) (American Red Cross Holland Laboratory, Rockville, MD)	Two outer layers of human fibrinogen (13.5 mg/cm^2^) and a middle layer of human thrombin (40 U/cm^2^) and CaCl_2_ (75 μg/cm^2^), freeze-dried onto an absorbable Dexon mesh backing [161]	Applied in a small pool of blood and compressed over the wound with sufficient pressure to occlude the vessel for 4 min in a swine aortic injury model
	Consisting of human fibrinogen, 0, 4, 8, 15 mg/cm^2^, human thrombin 37.5 U/cm^2^, and CaCl_2_, 40 mM/cm^2^, freeze-dried onto an absorbable polygalactin mesh backing [162]	Applied to the surface of the injured liver of swine with 60-s compression in the dorsoventral and lateromedial direction, respectively. Only the 15-mg formulation significantly reduced blood loss.
	Consisted of human fibrinogen, 10.3 mg/cm^2^, human thrombin, 28.6 U/cm^2^, and CaCl_2_, 2.5 mg/cm^2^, all compressed onto a silicone backing [163]	Applied in a uniform manner with 2 min of direct pressure and then tied in place with muslin backing in a swine model of ballistic injury
Fibrin sealant dressing (Fibriseal)	15 mg fibrinogen, 36 U thrombin, 3.6 mg CaCl_2_ /cm^2^ on a silicone backing [164]	Held in place on the femoral arteriotomy with a mechanical device in a swine femoral artery injury model
Fibrin Sealant Patch (Evarrest)	Oxidized regenerated cellulose, polyglactin 910 nonwoven fibers, human fibrinogen, and human thrombin [165]	Gauze packing with 3 min of standardized pressure in swine models of unilateral femoral artery puncture or a grade V liver laceration with timed free bleeding

Compared to liquid fibrin sealants, a solid patch/dressing can be applied with manual pressure to hold onto the bleeding site and allow cavity packing. It can be stored at room temperature, has a long shelf-life, and requires little preparation. However, solid fibrin sealants are more expensive and are hard to conform, making it difficult to seal a wound with an irregular surface [168, 169].

Clinical trials have shown the safety and efficacy of fibrin sealants for uncontrolled bleeding in trauma and across a variety of surgical types (normally in comparison with other products) [168, 170]. There is a renewed interest in fibrin sealant dressing composed of fibrinogen and thrombin in a dry form for severe bleeding in battlefield trauma given that its hemostatic efficacy is equivalent to Combat Gauze in swine models of unilateral femoral artery puncture or a grade V liver laceration with timed free bleeding [165]. This may also be because the fibrin sealant patch would have a clinical advantage over Combat Gauze and XStat since it is biodegradable and thus can be left in the body, thereby avoiding reoperation. It also acts independently of the clotting cascade; therefore, it may be more effective in the control of coagulopathic bleeding [169].

### Local hemostats under development

The current local hemostatic agents mainly belong to the group of hemostatic dressings and aim to help those with junctional hemorrhage. Since these topical agents need to be applied to bleeding sites with sustained manual pressure, they may not be suitable for care under fire scenarios [171]. Furthermore, pressure-required hemostats would not be useful for stopping intracavitary bleeding from the torso region involving noncompressible soft tissues/organs, e.g., pulmonary and torso vessels, grade 4 solid organs (liver, kidney, spleen) and open pelvic ring, an entity collectively labeled as “noncompressible torso hemorrhage” [172].

Safety concerns have also been raised for some local hemostatic agents [30, 31]. A recent survey indicated that hemostatic agents were infrequently utilized to manage traumatic hemorrhage during the recent conflicts in Afghanistan and Iraq [137]. A number of newer materials are under development for treatment of torso hemorrhage [19].

#### Flowable hemostatic agents

A typical example for this type of local hemostats is the thrombin-gelatin hemostatic matrix called Floseal, which has shown good safety and efficacy profiles in clinical studies compared to other flowable hemostats, e.g., microporous polysaccharide powder [173, 174]. Floseal may be the most studied flowable hemostatic agent. It consists of a gelatin matrix crosslinked by glutaraldehyde and ground to 500–600 μm particles and a thrombin solution (1000 U/ml) reconstituted with saline prior to use. The two components are mixed and applied to a wound using a special applicator syringe. On contact with blood, the gelatin granules swell and form intimate contact with damaged tissues to produce a tamponade effect and restrict blood flow. Furthermore, blood percolating through the gelatin matrix is exposed to high concentrations of thrombin, resulting in a stable clot independent of the coagulation cascade, which has been demonstrated in a swine renal injury model with hypothermic coagulopathy [175].

Floseal has been shown to be effective in a range of surgeries, such as cardiovascular surgery [174], hepatic surgery [176], and endoscopic sinus surgery [177]. A case report showed that Floseal provided effective hemostasis in partial splenectomy after penetrating trauma [178]. Floseal was also studied as a potential intracavitary hemostatic agent in a rat model of severe livery injury [179]. No prehospital use of Floseal has been reported.

Tranexamic acid-loaded starch microspheres may have potent hemostatic ability from the synergistic role of physical hemostasis by absorption of water to increase endogenous coagulation factors and platelets at the bleeding site and drug hemostasis by effective inhibition of fibrinolysis [180]. The drug-loaded material showed better hemostatic performance (shorter time to complete hemostasis and less bleeding volume) than a commercial hemostat made of microporous polysaccharide hemispheres in rabbit ear artery injury and liver injury, respectively.

Another representation of flowable hemostatic agents is the dry fibrin sealant powder called Fibrocaps (Raplixa; ProFibrix BV, Leiden, Netherlands) [181, 182]. The product is provided in 1 g in a vial and is easy to use and store. It consists of 79 mg of human plasma-derived fibrinogen and 726 U of human plasma-derived thrombin that has been separately spray-dried with trehalose to produce soluble, free-flowing microparticles. The trehalose coating prevents thrombin from converting fibrinogen to fibrin and causing clot formation when the mixture was prepared. Fibrocaps can be applied by direct sprinkling from the vial onto a bleeding site or spraying onto the bleeding site with an air-powered spray device called ProFibrix Fibrospray. The device was specifically designed to deliver Fibrocaps under air pressure. The system has several ongoing trials for the treatment of surgical bleeding across a wide range of surgery types, with demonstrated effectiveness in some trials [181–183].

#### Injectable hemostatic agents for local applications

Self-assembling peptides have shown unique hemostatic activities in in vitro and animal models [184–186]. One of the peptides is based on a 16-amino-acid self-assembling peptide, RADA16-I, consisting of an Arg-Ala-Asp-Ala motif repeated four times, and can be incorporated into common bandage and wound dressing materials via layer-by-layer assembly [187]. The peptide nanofibers elute upon hydration under physiological conditions and generate nanofiber-based clots with blood. Both in vitro clot formation and in vivo control of bleeding in porcine skin wounds demonstrated the use of the peptide in combination with commercial gauzes as a promising approach for an inexpensive yet effective hemostatic product. After exposure to a range of harsh temperature conditions (80 to 60 °C) for a week and even 5 months at 60 °C, these hemostatic bandages remain capable of releasing active nanofibers. Further studies in animal models of severe bleeding are still needed to test the efficacy of the peptide in an injectable form.

Cryogels composed of carbon nanotube and glycidyl methacrylate functionalized quaternized chitosan were developed as injectable hemostatic and antibacterial agents for lethal noncompressible hemorrhage and wound healing [188]. In vitro studies showed that these cryogels possessed robust mechanical strength, fast shape recovery, and instantaneous and high blood absorption capacity. Moreover, the cryogels showed better blood-clotting ability and higher blood cell and platelet adhesion and activation than commercial gelatin sponge and gauze. The cryogel containing 4 mg/mL carbon nanotube showed better hemostatic effect (less blood loss and shorter hemostatic time) than the gauze and gelatin sponge in a mouse liver injury model and a mouse tail amputation model and better wound healing performance than a commercial dressing (Tegaderm). Furthermore, the cryogel exhibited better hemostatic performance than the gelatin sponge in a rabbit liver defect lethal noncompressible hemorrhage model and even better hemostatic effect than Combat Gauze in a standardized liver bleeding model.

Surgical intervention is currently the only option for traumatic intra-abdominal hemorrhage, which is challenging given the variety of organs and vessels possibly ruptured or penetrated. A number of intracavitary foams are under development to stop bleeding from a noncompressible abdominal hemorrhage. One type is based on self-expanding polyurethane foams that expand rapidly and mold to organs to apply local pressure to the site of injury and seal wounded tissues [56, 189]. Peev [190] and Duggan [191] et al. described a percutaneously administered, self-expanding polyurethane foam that improved survival in a dose-dependent manner in a swine model of severe intra-abdominal hemorrhage. A polyol solution consisting of polyether polyol, polysiloxane surfactant, triethylenediamine catalyst, and an isocyanate solution were loaded into two sides of a dual barrel, polypropylene cartridge, and a pneumatic piston was used to dispense different volumes of each solution through a dynamic mixing nozzle specifically engineered for this purpose [191]. The foam was generated by a reaction between the two liquids within 2 min after injection into a peritoneal cavity. Mesar et al. [192] confirmed human dosage by injecting various volumes of the liquid precursors in recently deceased humans with representative tissue compliance. Although polyurethane foams appear promising, the major drawback is that they must be removed, and the hemorrhage typically resumes immediately. A clinical trial is underway to demonstrate the safety, effectiveness and benefit-risk profile of one type of polymeric foam, called ResQFoam, for the in-hospital treatment of emergent, exsanguinating, intra-abdominal hemorrhage resulting in Class III or IV hemorrhagic shock due to trauma (https://clinicaltrials.gov/ct2/show/NCT02880163).

Another type of intracavitary foam is based on a fibrin sealant embedded in a biomimetic complex polymer [193]. The so-called ClotFoam is a gelatin-based hydrogel carrying a fibrin monomer and produced from a mixture of four liquid components (e.g., gelatin, transglutaminase enzyme, fibrin monomer, factor XIII) [193]. It is currently an investigational product under a phase 1 clinical trial for hemostasis in liver bleeding [194]. When delivered through a CO_2_ propellant, the mixed solutions form a foamy gel that spreads throughout a body cavity to promote adhesion and stimulate the coagulation cascade. The foam enhances the distribution of active clotting agents and provides a scaffold upon which the fibrin network can be distributed and thereby adhere and bind to bleeding tissue. Given the noninvasive application and dissemination of the agent, ClotFoam may achieve hemostasis in the peritoneal or other body cavities without compression and/or sutures outside the operating room.

In addition to local hemostatic agents, devices have also been developed for prehospital hemorrhage control by applying mechanical forces [18]. For example, a device called iTClamp seals the skin edges within a pressure bar, enabling creation of a hematoma where blood collects under pressure to form a stable clot until definitive surgical repair [195]. The device was successful controlling junctional bleeding in a lethal swine exsanguination model. It has also been used in conjunction with Combat Gauze or XStat to treat arterial injuries through 5- or 10-cm skin incisions in the groin, axilla, or neck in a swine model [196].

Resuscitative endovascular balloon occlusion of the aorta using a compliant balloon has been used in a variety of clinical settings as an adjunct in torso hemorrhage, with limited success [197, 198]. The procedure is complex and may not be feasible for hemorrhage control in prehospital settings [199].

New tourniquet devices have been developed for abdominal and junctional hemorrhage and have been shown to be effective in stopping arterial flow and controlling bleeding in preclinical trials, but clinical data are lacking [200].

### Comparison of hemostatic agents

Clinical trials have been conducted to compare fibrinogen concentrate to cryoprecipitate [201] and fresh frozen plasma [93, 202], suggesting equivalent or improved clinical outcomes from the fibrinogen treatment. Freeze-dried plasma FLYP reduced the transfusion time, achieved a higher fibrinogen concentration and a greater improvement in prothrombin time, factor II and factor V compared to fresh frozen plasma in trauma patients [71]. No direct comparison among coagulation factor concentrates and fresh frozen plasma was found.

Numerous studies have been conducted to compare local hemostatic agents mainly in animal hemorrhagic models [20, 203, 204]. Swine models of femoral arterial or mixed arterial and venous hemorrhage and an extremity injury with mixed arteriovenous hemorrhage and a large soft tissue component have mostly been used in the recent comparative studies of operationally deployed hemostatic agents [205, 206]. Goat, rat and sheep models looking at arterial and/or venous bleeding were also used. Swine liver and spleen injury models have been developed to assess hemostatic agents for control of abdominal bleeding [191, 207].

Arnaud et al. [208] compared most (10) hemostatic dressings in groin injury (puncture and transection) models in swine. Based on survival and rebleeding rates, Celox WoundStat, X-Sponge, and QuikClot ACS+ showed better hemostatic efficacy than Dextran/chitosan, HemCon, ChitoFlex and BloodStop (deoxidized cellulose-coated gauze). In a swine model of groin injury with limited vessel access, standard gauze dressing, ChitoFlex dressing, QuikClot ACS dressing, and Celox dressing were evaluated for survival and hemostasis [209]. All performed well in mitigating blood loss and promoting survival, with no statistically significant differences.

Four hemostatic dressings currently deployed in combat operations were evaluated in femoral and axillary injury models created in extremities of live goats or pigs: Celox, ChitoGauze, Combat Gauze and HemCon wafer [210]. Overall, there were no significant differences in hemostasis and volume of blood loss between the dressings. Although not statistically significant, Combat Gauze achieved the highest hemostasis, at 83% by 4 min. Combat Gauze was also rated as the easiest dressing to use by the soldiers who participated in the study.

In a similar standardized swine model of femoral artery bleeding, Celox Trauma Gauze, Celox Gauze, and HemCon ChitoGauze performed at least as well as Combat Gauze in terms of survival rate, immediate hemostasis and blood loss, suggesting that contemporary hemostatic dressing technology has potentially reached a plateau for efficacy [211].

XStat and Combat Gauze were comparatively evaluated in a coagulopathic swine model of lethal junctional hemorrhage (transection of both the axillary artery and vein and replacement of 60% of blood volume with colloid solution) [212]. XStat performed better than Combat Gauze in terms of time to achieve and retain hemostasis and blood loss volume during the first 10 min after injury. However, there were no differences in survival.

In a swine femoral artery injury model with coagulopathy induced by 50% isovolemic hemodilution and hypothermia, two mineral-based agents, one in granular form composed of an alumino-silicate smectite mineral and an extremely water-absorbent polyacrylic acid salt (WoundStat) and the other in the form of gauze impregnated with kaolin (Combat Gauze), were compared with a fibrinogen-based (FAST) dressing [213]. FAST dressing showed the highest efficacy because of the exogenous delivery of concentrated fibrinogen and thrombin to the wound, which bypasses coagulopathy and secures hemostasis.

A systematic review identified seven prehospital hemostatic dressings for trauma (Celox granules, Celox gauze, ChitoGauze, HemCon bandage, QuikClot, QuikClot Advanced Clotting Sponge (ACS)+, Combat Gauze), with Combat Gauze being the most frequently applied in humans [54]. Kheirabadi evaluated five topical hemostatic agents (QuikClot ACS+, HemCon bandage, Celox powder, WoundStat, and Combat Gauze) for combat wound treatment [214], recommending that Combat Gauze may be sufficient for controlling the majority of compressible bleeding in noncoagulopathic patients, and fibrinogen-based dressings may offer the best chance for stopping bleeding in coagulopathic patients in prehospital and hospital settings after significant blood loss and fluid resuscitation.

All current field-deployed topical hemostatic agents rely on manual pressure to stop bleeding. This is not ideal in an under-fire combat setting or for torso hemorrhage. There is the greatest need for controlling bleeding from the aforementioned noncompressible torso wound, which is the leading cause of potentially preventable death in both civilian and military trauma [172].

There are no head-to-head comparisons between different topical hemostatic agents in bleeding patients. Zhang et al. [203] reviewed 41 studies of topical hemostatic agents (4 human case reports and 37 comparative animal studies) and recommended fibrin sealant dressing, Celox and WoundStat for life-threatening extremity hemorrhage on the battlefield.

A standardized swine femoral artery injury model has been developed [215] and is now widely used as a standard for preclinical studies and comparison of hemostatic agents. Although pigs with severe hemorrhage provide a validated hemorrhage model, they do not account for the combat conditions under which prehospital medical providers apply hemostatic agents. The current swine models with coagulopathic bleeding are created by either hemodilution or hypothermia and may not reflect trauma-induced coagulopathy in patients involving massive tissue damage [216].

Gauze-type dressings are preferred over powdered or granular hemostatic agents in combat wounds with bleeding from a narrow tract, as the former is more easily packed into the depths of such wounds and thus can make direct contact with the bleeding vessel [217]. Moreover, the powdered or granular agents may present an ocular hazard if used in a windy environment or in the presence of rotor wash from helicopters.

Systemic hemostatic agents promote hemostasis through biological mechanisms targeting different coagulation and fibrinolysis pathways. Compared to systemic hemostats, local agents exert hemostatic functions through biological as well as physiochemical and mechanical means and can be applied in different forms – as liquid, solid powder and sheet. The local hemostatic agents can stop bleeding based on the following mechanisms of action [205]: (A) factor concentrators (e.g., WoundStat); (B) mucoadhesive agents (e.g., Celox, HemCon); (C) procoagulant supplements (e.g., Combat Gauze, fibrin sealants); and (D) compression (e.g., XStat). Some agents can combine procoagulant, absorptive and tamponade effects to render efficient hemostasis.

## Safety

The main safety issues with systemic hemostatic agents include thromboembolic complications, transmission of blood-borne pathogens, and antibody response [218]. The risk of infection is now minimal as a result of effective pre- and postdonation screening that tests for potential pathogens and the institution of pathogen reduction strategies to which many plasma-derived biological medicines are now routinely subjected.

Prehospital administration of TXA during aeromedical transport was found to increase the risk of venous thromboembolic events compared with administration at the emergency department [111]. It has been recommended that a one-gram prehospital TXA bolus be administered in high-risk patients followed by subsequent doses guided by thromboelastography [42]. Additionally, a pharmacovigilance study concluded that there was no increased risk of thromboembolic events in FC-treated patients [219].

Despite these great successes, there are safety concerns with the prehospital or in-combat use of local hemostatic agents [30, 135, 205]. Although no adverse events have been reported in human studies with the use of Combat Gauze, HemCon, Celox Gauze, Celox granules, ChitoGauze and QuikClot ACS+ [54, 204], these studies are relatively short-term observational studies. Animal studies raised concerns with the presence of intraluminal particles and thrombi as well as tissue damage 2 to 3 h after treatment when WoundStat, super quick relief (another mineral-based hemostat composed of potassium iron oxyacid salt and hydrophilic polymer) and Celox were compared against HemCon, QuikClot ACS+ and Combat Gauze [31, 220]. Otrocka-Domagała et al. [30] observed macroscopic and microscopic severe changes and shock symptoms in the lungs, liver, kidneys and heart and fibrino-gaseous embolic material in the pulmonary artery and in the lung vessels 24 h after treatment with Combat Gauze, ChitoGauze and Celox Gauze in a femoral artery injury model in swine. The risk of the transmission of infectious agents may be another problem associated with the application of biological hemostatic agents [221].

## Conclusions

Systemic agents can be administered to staunch bleeding at inaccessible injury sites, which may impose a large challenge for local agents limited to treating visible and accessible injuries. However, a major concern with the use of systemic agents is the potential for subsequent thromboembolic complications, as they may act on coagulation and fibrinolysis pathways in the circulatory system. Dried blood products have logistic advantages over their liquid counterparts in a prehospital setting, especially in an austere combat environment.

Combat Gauze, HemCon and Celox are three major product brands investigated for combat and prehospital hemorrhage control, with considerable preclinical and clinical evidence for their effectiveness, but there is inconclusive evidence to differentiate one product as more effective than others. Extensive research has been conducted to evaluate and compare the effectiveness of topical hemostatic agents for the control of severe hemorrhage in a variety of animal models. The majority of these animal studies were swine models with groin injuries and found the three types of agents to be equally effective.

There is a paucity of published clinical literature that provides high-quality evidence for the use of one type of hemostatic dressing over another in humans through randomized controlled trials [54]. With that in mind, Combat Gauze may be justified as the currently optimal topical hemostatic dressing due to having the most clinical data and its safety profile, although all studies were at risk of bias and were assessed to be of ‘very low’ to ‘moderate’ quality [54].

Most local hemostatic agents may not work under conditions where the blood clotting mechanisms are impaired [54, 214], so-called coagulopathic bleeding. For example, Combat Gauze consists of nonwoven gauze impregnated with kaolin, which speeds up blood clotting. However, kaolin fails to work under conditions when the blood clotting mechanisms are impaired, thus preventing effective hemorrhage control [213].

In addition, the prehospital hemostatic dressings are mainly for external bleeding and may be inefficient for internal or intracavity bleeding. A literature review suggested the use of local pro-coagulant hemostatic agents, such as thrombin and fibrinogen, for the control of intracavity bleeding [169].

There are many new hemostatic agents under development, each with their own pros and cons based on the type of injury, severity of bleeding, wound size and configuration, location on the body, accessibility to the bleeding site, and the patient’s coagulation function. Each should be reviewed for their specific value added for patient care.

## Future directions

None of the current hemostatic products meet all criteria for an ideal hemostatic agent. There is still a tremendous impetus for the development of diverse and more efficacious hemostatic technologies and biomaterials for emergency scenarios, not only in both civilian and military traumatic settings but also during variable therapeutic interventions.

Alternatively, large randomized trials, particularly direct head-to-head clinical trials comparing the current hemostatic agents, are needed to show which is more effective in prehospital hemorrhage control. There is also a need for studies that would enable the selection of appropriate hemostatic agents based on the mechanism and location of injuries.

Finally, nanotechnology has been applied for hemostasis [222]. Some novel hemostatic agents based on nanomaterials are intriguing [223]; however, it may be some time before they are brought to clinical trials. For example, nano- and micromaterials have been developed for the treatment of internal bleeding and uncontrolled hemorrhage [223]; however, these novel hemostatic agents again may prove to be efficacious (or not) with further studies. There are also new delivery systems (e.g., self-driven and trauma-targeted delivery) that would make current hemostatic agents more effective [224, 225].

## Data Availability

Not applicable.

## Abbreviations

CFC: Coagulation factor concentrates
CRASH-2: Clinical randomization of an antifibrinolytic in significant hemorrhage 2
DFSD: Dry fibrin sealant dressing
FC: Fibrinogen concentrate
FDP: Freeze-dried plasma
FFP: Fresh frozen plasma
FLYP: French lyophilized plasma
LyoPlas: German lyophilized plasma
PCC: Prothrombin complex concentrate
RBC: Red blood cells
TF: Tissue factor
TXA: Tranexamic acid
WOMAN: World maternal antifibrinolytic
US: United States
